# Trajectories in tension: social capital, access to resources, and structural trials in Chile and Mexico

**DOI:** 10.3389/fsoc.2026.1758470

**Published:** 2026-05-08

**Authors:** Patricia Nieto-Rivera

**Affiliations:** Facultad de Humanidades, Universidad de Santiago de Chile, Santiago, Chile

**Keywords:** positional inconsistency trial, work trial, social capital, relational resources, structural equation modeling, Latin America

## Abstract

**Introduction:**

This article examines how ascribed and achieved factors influence exposure to structural trials in unequal contexts.

**Methods:**

A comparative quantitative design was applied using survey data from 300 economically active individuals in Chile and Mexico, analyzed through EFA, CFA, and SEM.

**Results:**

Social capital and relational supports significantly reduce both positional inconsistency and work trials, with notable cross-national differences.

**Discussion:**

Findings highlight the central role of relational resources in mitigating structural fragility and contribute to understanding stratification processes in Latin America.

## 1 Introduction

Latin America is characterized by high levels of inequality and persistent institutional fragility, conditions that shape individual trajectories and constrain opportunities for social mobility. In these contexts, access to material and relational resources is distributed in profoundly unequal ways, reinforcing processes of cumulative advantage and the reproduction of disadvantage (Espinoza and Otero, 2024; Carrascosa, 2024; Burt, 1992). Individual trajectories therefore depend not solely on merit, but on social structures that differentially allocate economic, educational, and social capital.

Regional stratification is also marked by long-standing histories of exclusion that complicate the role traditionally attributed to education as the primary mechanism of upward mobility (Barozet et al., 2021). In this setting, approaches capable of capturing the lived experience of inequality beyond conventional indicators become particularly relevant. The notion of structural trials, developed by Araujo and Martuccelli (2012), offers a framework for analyzing the tension between structure and biography by conceptualizing historically configured, socially produced, and unequally distributed challenges that individuals must confront.

This article focuses on two structural trials that are especially salient in highly unequal societies: positional inconsistency and the work trial. The former refers to the fragility of social positions and the fear of downward mobility, capturing concerns related to status and the need to mobilize support in order to sustain unstable positions. Although it resonates with notions such as the “fear of falling” proposed by Barbara Ehrenreich (1989) or “status anxiety” as discussed by Alain de Botton (2004), positional inconsistency is particularly suited to the Latin American context because it does not require explicit class identification and instead emphasizes the structural vulnerability of social positions. The work trial, in turn, examines how the weakened integrative capacity of employment—under conditions of widespread precariousness and informality—affects economic stability, social recognition, and meaning-making (Castel, 2004; Paugam, 2012). Together, these trials provide an analytical lens for understanding how individuals navigate uncertainty in contexts where merit does not guarantee stability or recognition.

While existing scholarship has demonstrated the relevance of structural trials for capturing relational and subjective dimensions of inequality, a gap remains regarding how ascribed and achieved factors differentially structure exposure to these trials in Latin America. In particular, it is necessary to examine the articulation between social origin, educational attainment, occupational status, social capital, and access to resources in order to identify patterns of reproduction or mitigation of social and labor-related fragility.

The aim of this article is to empirically assess the influence of ascribed variables (social origin) and achieved variables (educational attainment, social capital, occupational status, and access to resources) on positional inconsistency and the work trial. To this end, the analysis relies on data collected in Chile and Mexico, two highly unequal societies that share structural fragilities but display distinct social and labor configurations. The analytical model, developed in the methodological section, conceptualizes social origin as an exogenous variable structuring educational and relational trajectories, while acquired resources and capitals mediate exposure to both structural trials. This framework allows for a comparative assessment of how these relationships are configured across the two national contexts.

The article is organized into five sections: conceptual framework, methodological design, results, discussion, and conclusions. By linking structural trials with relational analysis, this study contributes to a context-sensitive understanding of contemporary stratification processes in Latin America.

## 2 Conceptual framework

### 2.1 Social challenges, inequality, and stratification

The sociology of “desafíos sociales” (social challenges), developed by Kathya Araujo and Danilo Martuccelli, offers a renewed analytical lens for understanding stratification in contexts marked by persistent inequality and institutional fragility, such as those found across Latin America. At the core of this approach lies the notion of “pruebas estructurales” (structural trials): historically shaped, socially produced, and unequally distributed challenges that individuals must confront throughout their life trajectories (Araujo and Martuccelli, 2012; Araujo, 2011, 2013). These trials do not merely refer to individual difficulties; rather, they reveal structural tensions linking social position, vulnerability, and action, thus enabling an examination of the lived experience of inequality beyond conventional indicators of stratification.

Drawing on C. Wright Mills’ sociological imagination and François Dubet’s sociology of experience (Dubet, 2006, 2010), this perspective starts from the diagnosis of growing deinstitutionalisation: institutions no longer provide stable guarantees of recognition, integration, or meaning (Dubet, 2006). In this context, individuals must engage in sustained self-management of their trajectories, mobilising personal and relational resources to maintain their position within the social hierarchy (Martuccelli and Santiago, 2007). Yet the capacity for such self-management is deeply conditioned by structures that distribute available resources unevenly, revealing not only the unequal distribution of trials but also the unequal distribution of the means to confront them.

From this perspective, trials are not experienced homogeneously. They refract through social origin, gender, cultural capital, relational resources, and individual biographies. Some individuals possess material, symbolic, or relational assets that allow them to manage fragility, while others lack sufficient support, reproducing inequalities at the level of lived experience (Araujo and Martuccelli, 2010). Inequality manifests not only in differential access to resources but also in unequal ways of facing everyday vulnerability.

Within this framework, the notion of “soporte” (support) becomes central. Supports refer to the heterogeneous set of elements—family ties, trust networks, institutional resources, or symbolic strategies—that individuals rely on when facing a trial (Araujo and Martuccelli, 2012). In Latin American contexts, where state and market protections are limited, family and close networks provide care, security, and recognition functions that, in other settings, are supplied by formal institutions (Valenzuela et al., 2006; Di Leo and Camarotti, 2017). Supports therefore mediate not only access to material resources but also the production of belonging and symbolic validation, which are essential for subjectivity in contexts of precarity.

While research on structural trials has primarily relied on qualitative methods—especially in-depth interviews that reconstruct the relationship between structure and biography (Araujo and Martuccelli, 2012; Navarro Leal and Sembler Reyes, 2018)—recent advances have sought to operationalise these categories through quantitative approaches. Such developments expand their explanatory potential and enable comparisons across social groups and national contexts.

Araujo and Martuccelli (2012) identify nine structural trials that concentrate some of the central tensions of contemporary social life: i) structural change, ii) democratisation, iii) social bonds, iv) positional inconsistency, v) temporal imbalances, vi) work, vii) merit, viii) relational irritations, viiii) and family. Each trial captures a specific dimension of vulnerability and action, providing insight into how individuals confront the imperatives and constraints of their historical moment.

This article focuses on two of these trials: the work trial and the positional inconsistency trial. The work trial illuminates conditions of labour-market insertion—formality, stability, and recognition—that shape life opportunities and reproduce inequalities linked to class, gender, and social origin. The positional inconsistency trial addresses the misalignments between education, occupation, and income, revealing the fractures of the meritocratic promise and the fragility of social positions. Together, these trials shed light on how stability and recognition in highly unequal Latin American societies depend not only on individual achievements but also on the supports individuals are able to mobilise when facing uncertainty.

### 2.2 The trial of positional inconsistency

The prueba de la inconsistencia posicional (trial of positional inconsistency) offers an analytical lens for understanding status fear, conceived as a set of experiences marked by concerns over one’s place in the social hierarchy. In the literature, this form of fear has been addressed primarily through three perspectives: fear of falling, status anxiety, and positional inconsistency.

#### 2.2.1 Fear of falling

The concept of fear of falling, developed by Barbara Ehrenreich (1989), describes the anxiety experienced by the U.S. middle class in relation to the possibility of losing status and undergoing socioeconomic decline. This fear emerged in a context of growing criticism of the educational system—historically the main mechanism of social reproduction—which cast doubt on the stability of professional positions.

Beyond its specific historical origin, fear of falling denotes a collective unease triggered by the perceived threat of losing privileges and witnessing the erosion of class boundaries. This concern is often linked to the perception of an “upward invasion” by less advantaged groups (Aarseth, 2017) and manifests as a group-oriented anxiety aimed at protecting advantages and status (Jetten et al., 2017). In essence, it reflects a shared sense of threat that exposes the symbolic fragility of middle-class identities.

#### 2.2.2 Status anxiety

Status anxiety, as formulated by Alain de Botton (2004), refers to the concern of not receiving the expected esteem or social recognition. Unlike Ehrenreich’s approach, De Botton conceptualizes status as a symbolic value tied to admiration and public acknowledgment.

This anxiety arises when individuals fear failing to meet culturally defined standards of success—often framed in terms of material or professional achievements—and interpret this gap as failure or humiliation. In highly competitive societies, such anxiety is intensified by constant comparison with others and is experienced in a private, often silent manner. Unlike fear of falling, status anxiety is fundamentally individual and closely connected to the pursuit of personal validation.

#### 2.2.3 Positional inconsistency

The notion of “inconsistencia posicional” (positional inconsistency), developed by Araujo and Martuccelli (2012), shifts the focus from stratification mechanisms to the subjective experience of positional fragility. This perspective examines how individuals perceive the instability of their social positions—particularly the possibility of downward mobility—in contexts where access to resources, goods, and opportunities is not guaranteed (Araujo, 2011).

Positional inconsistency captures the vulnerability that arises when individuals cannot secure the resources needed to sustain a given position. This experience may stem from economic instability, labor precarity, urban insecurity, or political changes (Arteaga and Martuccelli, 2011), and manifests along a continuum: some experience it as occasional unease, while others confront it as a persistent threat. In response, individuals mobilize various forms of support—familial, community-based, or institutional—to stabilize their social positions (Martuccelli, 2007).

Unlike fear of falling, centered on the collective defense of group privileges, positional inconsistency cuts across social classes and emerges through individualized experiences. This breadth makes it particularly relevant for Latin America, where class consciousness is diffuse and social trajectories are unstable (Archila, 1989). It also differs from status anxiety: whereas status anxiety turns social ties into sources of distressing comparison, positional inconsistency emphasizes the role of others as critical supports that help individuals navigate fragility (Araujo and Martuccelli, 2012; Navarro Leal and Sembler Reyes, 2018; Di Leo and Camarotti, 2017).

In Latin American contexts, where formal employment does not reliably provide social protection, family ties and close networks play decisive roles in offering economic, emotional, and care-related support (Valenzuela et al., 2006). For this reason, using positional inconsistency as a lens to analyze status fear is more appropriate than relying on status anxiety alone, as it captures the structural and relational fragility shaping social positions across the region.

In sum, whereas status anxiety tends to emerge in societies where social positions are relatively stable and individual success is framed as a moral imperative, positional inconsistency characterizes contexts of structural uncertainty—settings in which social positions themselves are perceived as fragile and shifting, and where individuals must continually reaffirm their place within the social order.

### 2.3 Work as a structural trial

Work has historically held a central place in the organization of modern societies, linking economic integration, collective belonging, and symbolic recognition. Within the sociology of social challenges, it constitutes one of the most transversal structural trials, condensing the effects of productive transformation and the fragility of traditional mechanisms of integration (Araujo and Martuccelli, 2012).

Among the theoretical approaches that conceptualize work as a trial, the contributions of Robert Castel and Serge Paugam are particularly relevant. Castel (1998, 2004, 2016) situates his reflections within the decline of the wage-based society, understood as the regime that, for much of the twentieth century, connected stable employment, social rights, and citizenship. The erosion of the welfare state, increasing labour flexibilization, and the expansion of atypical employment forms have weakened this model, producing processes of disaffiliation characterized by the loss of guarantees, economic instability, and exposure to new forms of insecurity. In this context, work becomes a challenge: individuals must continuously reaffirm their value and sustain their integration without the institutional supports that once provided stability.

Complementarily, Paugam (2012) distinguishes between employment precariousness and work precariousness. The former refers to insecurity generated by flexibilization and the weakening of labour protections; the latter concerns the deterioration of the labour experience itself, marked by work intensification, loss of meaning, and symbolic devaluation. Both dimensions require workers to demonstrate their usefulness on a permanent basis, exposing them to risks of social disqualification—an erosive process that undermines identity and weakens collective bonds.

Although Castel and Paugam converge in diagnosing the weakening of employment as an institution of integration, they differ in their analytical focus: Castel emphasizes structural transformations in the wage-based regime, while Paugam highlights the subjective consequences of deteriorated labour conditions. This distinction helps trace the shift from a model of integration anchored in employment stability to one in which work becomes a source of vulnerability.

Araujo and Martuccelli (2012) build on and expand these discussions, arguing that the trial of work expresses, in a particularly pronounced way, the tensions characteristic of Latin American societies (Araujo, 2016). Labour flexibilization, permanent competition, and the erosion of the collective meaning of work generate a scenario in which individuals must manage their own stability and sense of belonging. Within this landscape, the experience of work disproportionality synthesizes the demands of contemporary productive systems: overload, uncertainty, pressure for performance, and the need to sustain personal meaning in precarious environments.

The authors identify three analytical dimensions of this trial. The first is pluriactivity, referring to fragmented labour trajectories, whether due to successive job changes or the simultaneous combination of multiple occupations. These trajectories give rise to neo-careers, intentional paths, or inertial trajectories that reveal the disconnection between work, professional identity, and stability. The second dimension concerns work irritations: everyday tensions among coworkers in contexts of competition, outsourcing, or weakened cooperation, which generate discomfort without structurally altering the employment relationship. The third dimension is meaning-making, referring to individuals’ efforts to attach value and purpose to work as a space of fulfillment and recognition (Dubet, 2006, 2010), even when it unfolds under increasingly unstable and demanding conditions.

Unlike Castel’s and Paugam’s perspectives, the sociology of social challenges emphasizes that, in Latin America, social integration does not depend exclusively on employment or its stability. Because work has historically failed to provide either protection or recognition, family ties play a fundamental role as supports in contexts of vulnerability. In this sense, the trial of work highlights the centrality of these supports for sustaining social trajectories and for giving meaning to labour in settings marked by precarization.

In sum, conceiving work as a structural trial makes it possible to understand how precarization simultaneously affects economic security, identity, and social ties. Together with positional inconsistency, this trial illuminates the lived experience of inequality and shows how social trajectories unfold at the intersection of structure, biography, and relational supports—particularly in Latin American contexts where uncertainty is a structural feature of working life.

### 2.4 Social capital: a generator of positions and resources

The concept of social capital has acquired multiple meanings within the social sciences, producing a certain degree of ambiguity and turning it into a “black box” (Portes, 2000). It is therefore essential to specify how social capital is understood in each study. In this article, it is conceived as the potential embedded in social relations, ties, and sociability to become sources of resources, power, and influence. From this perspective, social capital operates as a resource comparable to money insofar as it facilitates access to goods and opportunities (Bourdieu, 2000).

Although social capital manifests itself through interactions and networks, its analytical relevance lies in its status as a resource. As an asset, it complements natural, physical, financial, and human resources, distinguishing itself by residing within social relations (Raczynski and Serrano, 2005). This relational dimension makes it indispensable for understanding why individuals gain unequal access to opportunities even when they possess similar material resources (Bargsted et al., 2020).

The theoretical development of the concept has been structured around two main approaches: the associative approach, and the instrumental or individual approach. This study adopts the latter, which conceives social capital as a form of investment that yields individual returns. From this standpoint, people participate in networks that grant them access to information, the ability to influence others, and the reinforcement of identity and recognition (Lin, 2001; Bourdieu, 2000; Espinoza et al., 2017).

Authors such as Bourdieu, Burt, and Lin argue that social capital provides benefits that shape the acquisition and maintenance of positions within the social structure; that is, individuals may obtain advantages derived solely from their relationships (Lin and Dumin, 1986). However, access to this resource is unequal and depends both on the composition of networks and on individuals’ capacity to manage and mobilize them (Espinoza and Rabi, 2009).

The instrumental approach has developed multiple methodological tools to measure social capital. Among the most widely used are Lin and Dumin’s (1986) position generator and De Gaag and Snijders’s (2005) resource generator. The former assesses access to different social positions through the occupations of one’s contacts and the nature of the tie (strong or weak) (Lin and Erickson, 2008). The latter identifies the concrete resources available through networks—such as emotional support, financial assistance, or job information—thus enabling an estimation of social capital that extends beyond the occupational status of contacts.

As shown in previous sections, social capital plays a central role in how individuals confront the structural trials of positional inconsistency and the trial of work. This study argues that understanding these processes requires relying on the tools of the instrumental approach, in order to specify how social capital shapes the perception and navigation of these trials, in articulation with other factors such as education and social origin.

## 3 Methodological design

This study adopts a quantitative approach to analyze how ascribed and achieved factors influence two structural trials in contexts of high inequality: positional inconsistency and the work trial. The research is conducted in Chile and Mexico, two countries characterized by high levels of inequality and labor market heterogeneity yet displaying significant differences in their welfare regimes and occupational structures (Vargas, 2019).

The combination of structural similarities and institutional contrasts makes both cases suitable settings for examining the operation of these trials and assessing how inequality shapes the trajectories of individuals embedded in relatively stable occupations but exposed to region-specific structural tensions.

To operationalize both trials—typically examined through qualitative methodologies—a structured questionnaire was designed, incorporating a position generator and a resource generator. The instrument was previously pilot-tested, although the details of this process are not discussed in this article, in order to refine item formulation and ensure their conceptual and contextual adequacy.

### 3.1 Instrument

The instrument was designed to operationalize positional inconsistency and the work trial, while also incorporating ascribed and achieved factors in order to assess their influence on these trials. Its development was grounded in a review of the specialized literature on structural trials, as well as prior research on social capital and relational resources.

The latent variables included in the analytical model were operationalized through 30 observed indicators. The work trial was measured using 7 items, positional inconsistency through 11 items, and access to resources through 8 items derived from the resource generator. Social origin and social capital were each incorporated using two indicators. In the case of social capital, the indicators—network diversity and average network status—were constructed from the position generator, synthesizing information collected on 14 occupations^1^.

Table 1 presents the detailed description of each indicator and its associated measurement scale, as well as its correspondence with the model’s latent variables.

**Table 1:** Measurement model: latent constructs and indicators.

Latent variable	Observed variable	Response scale
Work trial	At work, I can freely express my opinions without fear of being dismissed.	Measured using a 5-point Likert scale capturing the degree of agreement with each statement (1 = strongly disagree, 5 = strongly agree). Higher scores indicate a more positive evaluation of work and, therefore, a lower presence of the trial.
	There is equal and respectful treatment in the workplace.	
	If I have a problem, I receive support from my supervisors.	
	I frequently talk with my coworkers about how to carry out the work.	
	If needed, I would receive help from my coworkers.	
	There is an atmosphere of trust among coworkers.	
	I feel that I am part of a work group or team.	
Positional inconsistency trial	You or a member of your family losing their job.	Measured using a 5-point Likert scale assessing the level of concern regarding different scenarios (1 = not concerned at all, 5 = very concerned). Higher scores reflect greater concern and, therefore, a stronger perception of this structural trial.
	Not having a place to live in the near future.	
	Not being able to pay bills in the coming months.	
	Not having enough money to purchase necessary food.	
	Not having money to cover potential medical treatment.	
	Having to change your lifestyle because you cannot afford it.	
	Becoming over-indebted.	
	Not being able to find jobs offering better working conditions than your current one.	
	Experiencing unfair treatment.	
	Losing the esteem, recognition, and support of others.	
	Experiencing some form of violence.	
Access to resources	If I needed money, a family member could lend it to me.	Measured using a 5-point Likert scale assessing the perceived availability of material and relational support through family and friendship ties (1 = strongly disagree, 5 = strongly agree). Higher values indicate greater resource availability.
	If I needed a vehicle, I could turn to a family member.	
	If I needed a vehicle, I could turn to a friend.	
	If I or a member of my household suffered a serious illness, a family member would provide care.	
	If I or a member of my household suffered a serious illness, a friend would provide care.	
	If needed, a family member or friend would help with childcare and/or care for older adults.	
	If I did not have a place to sleep, a family member could accommodate me temporarily.	
Social capital	Network diversity	Number of occupational positions in which the respondent reports knowing someone. For the modeling stage, the originally continuous variable was transformed into an ordinal categorical variable with three levels: low, medium, and high diversity.
	Average occupational status of the network	Mean occupational status of the known positions. For the modeling stage, the originally continuous variable was transformed into an ordinal categorical variable with three levels: low, medium, and high occupational status.
Social origin	Mother’s highest educational attainment	Constructed based on parents’ educational attainment, grouped into the following categories: ISCED 1: Incomplete primary; ISCED 2: Complete primary; ISCED 3: Incomplete secondary; ISCED 4: Complete secondary; ISCED 5: Tertiary (professional and postgraduate).
	Father’s highest educational attainment	

Source: authors’ elaboration, 2025. To compare parents’ educational attainment in Mexico and Chile, the ISCED (International Standard Classification of Education) classification was used, which harmonizes educational systems into five equivalent levels.

### 3.2 Sample

Data were collected using a non-probability convenience sampling strategy, consistent with the analytical focus of the study, which prioritizes the estimation of structural relationships rather than population-level inference. Accordingly, sample size was determined based on adequacy criteria for confirmatory factor analysis (CFA) and structural equation modeling (SEM), emphasizing model identification and estimation stability.

With 30 observed indicators, efforts were made to maintain a case-to-parameter ratio within the ranges commonly recommended in the methodological literature (5–10 cases per parameter), ensuring adequate convergence and sufficient statistical power to detect effects of moderate magnitude (Palacios and Vargas, 2009).

The selection of employed individuals earning above the minimum wage and residing in the main economic capitals responds to the substantive objective of the study: to evaluate the functioning of the structural model among relatively stable labor segments embedded in highly unequal urban contexts^2^.

Two independent samples were obtained:

- *Mexico city*: 200 cases collected through telephone surveys administered by trained interviewers.
- *Metropolitan region of santiago*: 100 cases gathered through an online questionnaire disseminated via channels targeting the intended profile.

The Mexican sample (*n* = 200) achieves statistical power above 0.80 for detecting moderate effects, following criteria reported by Wolf et al. (2013). Although smaller, the Chilean sample (*n* = 100) meets minimum thresholds for model estimation and allows for assessing structural consistency in a second national context^4^. Taken together, both samples are adequate for testing and comparing the proposed structural model.

### 3.3 Sample characteristics

The main sociodemographic, educational, occupational, and income characteristics of both samples are presented below. This information provides contextual grounding for the subsequent factorial and structural analyses and allows for assessing cross-national comparability as well as internal heterogeneity relevant to the interpretation of the structural trials.

#### 3.3.1 Gender

In Mexico, 49.2% of participants identified as female, 49.7% as male, and 1% as non-binary. In Chile, women represented 52.7% and men 49.2%.

#### 3.3.2 Education

In Mexico, 86.9% of respondents have tertiary education, and of these, 27.3% hold a postgraduate degree. In Chile, these figures rise to 95.5 and 58.9%, respectively, reflecting a higher concentration of advanced credentials in the Chilean sample.

#### 3.3.3 Occupation

Both samples are concentrated in scientific and intellectual professions, with a higher proportion in Chile (67%) than in Mexico (58.4%). Mexico shows a greater presence of managers and executives, while Chile has more technicians and associate professionals (see Table 2).

**Table 2:** Occupational group.

Occupational group	México (%)	Chile (%)
Professionals scientific and intellectual	58.4	67.0
Managers and executives	19.0	7.1
Technicians and associate professionals	10.2	17.9
Administrative support personnel	7.1	4.5
Service and sales workers	4.6	0.9
Elementary occupations	0.5	2.7

Source: authors’ elaboration, 2025.

#### 3.3.4 Income

Income distributions are concentrated in middle ranges. Chile has a higher minimum wage, which affects relative comparisons. Lower and upper brackets are less frequent in both countries (see Table 3).

**Table 3:** Income intervals in USD among respondents.

Income interval (USD)	México (%)	Income interval (USD)	Chile (%)
Up to $230	0.5		
$230.20—$460.50	3.5	Up to $565	2.7
$460.60—$690.90	16.7	$566—$770	16.1
$680.70—$1152.50	23.7	$770—$990	17
		$990—$1.320	16.1
$1152.60—$2303.60	37.9	$1.320—$1.870	19.6
		$1.870—$2.200	7.1
More than $2.303.70	17.2	More than $2.200	20.5
Missing	0.5	Missing	0.9

Minimum wage CDMX 335.87 USD (2023).

Minimum wage Chile 566.03 USD (2025).

Source: authors’ elaboration, 2025.

### 3.4 Reliability of the instrument

Prior to implementing the analytical strategy, the internal reliability of the instrument was assessed separately for each country. In Mexico, the full set of items showed high internal consistency, with a Cronbach’s alpha of 0.86 and a G6 coefficient of 0.92. Additionally, the omega total reached 0.89, indicating that a substantial proportion of the observed variance is attributable to common factors. However, the omega hierarchical was 0.30, suggesting that only a limited portion of the variance can be attributed to a general factor, which is consistent with a predominantly multidimensional structure.

A highly similar pattern was observed in Chile. Cronbach’s alpha was 0.85 (95% CI: 0.81–0.89), the G6 coefficient reached 0.94, and the omega total was 0.89, confirming high overall reliability of the item set. The omega hierarchical was 0.27, again indicating that the common variance is primarily distributed across multiple specific dimensions rather than a single dominant general factor.

Taken together, these results provide robust evidence of internal consistency in both samples and support the reliability of the instrument as a multidimensional measure. The convergence of the reliability coefficients across countries suggests comparable psychometric performance of the instrument in both contexts, supporting its suitability for comparative structural analyses, subject to the formal verification of factorial invariance.

### 3.5 Analytical strategy

The analytical strategy was conducted in four complementary stages. First, descriptive statistics and mean comparison tests (Student’s t-test) were estimated to identify cross-national differences in the model’s main indicators.

Second, an Exploratory Factor Analysis (EFA) was conducted to identify the latent structure of the indicators and assess the instrument’s internal coherence. Based on these results, adjustments were made to optimize the questionnaire for online implementation.

Third, Confirmatory Factor Analysis (CFA) models were estimated to validate the factorial structure and evaluate model fit in each country.

Finally, Structural Equation Models (SEM) were implemented to estimate the relative influence of ascribed and acquired factors on the structural trials, as well as the correlations between them.

All analyses were conducted sequentially in both samples, applying the same analytical procedure in Mexico and Chile.

## 4 Results

### 4.1 Descriptive results

This section presents the descriptive and comparative results for the model’s latent variables: work trial, positional inconsistency trial, access to resources, and social capital. Descriptive statistics and between-country comparisons are reported for Mexico and Chile.

#### 4.1.1 Work trial

Table 4 presents the mean scores, standard deviations, and mean differences for the items composing the latent dimension “work trial,” where lower scores indicate greater perceived intensity.

**Table 4:** Descriptive statistics and mean differences for work trial items (Mexico and Chile).

Item	Mexico mean	Mexico SD	Chile mean	Chile SD	*t*	*p*
At work I can freely express my opinions without fear of being fired.	4.07	1.16	3.90	1.23	1.19	0.236
At work there is equal and respectful treatment.	4.24	1.01	4.00	1.13	1.90	0.058
If I have a problem, I receive help from my supervisors.	3.24	1.05	2.97	1.02	2.12	0.035*
I frequently talk with my coworkers about how to carry out the work.	4.23	1.04	4.01	1.10	1.78	0.076
If needed, I would receive help from my coworkers.	4.38	0.87	4.07	1.09	2.77	0.006**
There is an atmosphere of trust among coworkers.	4.21	1.01	3.81	1.04	2.16	0.031*
I feel that I am part of a work group or team.	4.28	0.97	4.04	1.11	2.00	0.047*

Scale ranges from 1 (Strongly disagree) to 5 (Strongly agree).

*p < 0.05; **p < 0.01.

Source: authors’ elaboration, 2025.

In both countries, mean levels reflect generally positive evaluations of the work environment. However, systematic cross-national differences emerge. Across all indicators, mean scores are consistently higher in Mexico, suggesting a comparatively lower perceived intensity of the work trial relative to Chile.

Independent samples t-tests reveal statistically significant differences in several dimensions of relational support at work. Mexican respondents report significantly higher levels of supervisor support, coworker support, workplace trust, and sense of belonging to a work group. Differences in perceived equal treatment and communication with coworkers approach conventional levels of statistical significance, while no significant difference is observed in the freedom to express opinions at work.

Overall, these findings indicate that the work trial is experienced with greater intensity in Chile, whereas in Mexico more favorable evaluations of the work environment and interpersonal support tend to prevail.

#### 4.1.2 Positional inconsistency trial

Table 5 presents the mean scores, standard deviations, and mean differences for the items measuring positional inconsistency, where higher values indicate a greater perceived risk of losing one’s social position.

**Table 5:** Positional inconsistency trial items: means, standard deviations, and *t*-tests by country.

Item	Mexico mean	Mexico SD	Chile mean	Chile SD	*t*	*p*
That you or a family member might lose your job.	3.11	1.34	3.97	1.17	−5.68	<0.001***
Not having a place to live in the near future	2.63	1.66	3.56	1.56	−4.87	<0.001***
Not being able to pay bills in the coming months	2.77	1.49	3.70	1.45	−5.29	<0.001***
Not having enough money to buy necessary food	2.42	1.63	3.47	1.58	−5.46	<0.001***
Not having money to cover possible medical treatments	3.40	1.51	3.99	1.25	−3.66	<0.001***
Having to change your lifestyle because you cannot afford it	2.63	1.33	3.12	1.34	−3.18	0.002**
About becoming over-indebted	2.81	1.51	3.68	1.33	−5.03	<0.001***
Not being able to find a job that offers better working conditions than the current one	3.07	1.43	3.67	1.30	−3.63	<0.001***
Suffering unfair treatment	3.14	1.35	3.60	1.35	−2.88	0.004**
Losing the esteem, recognition, and support of others	2.62	1.34	3.31	1.35	−4.26	<0.001***
Experiencing some type of violence	3.55	1.39	3.75	1.49	0.67	0.501

Scale ranges from 1 (It does not worry me) to 5 (It worries me a lot).

*p < 0.05; **p < 0.01; ***p < 0.001.

Source: authors’ elaboration, 2025.

Across all items, mean scores are systematically higher in Chile, indicating a comparatively stronger perception of positional insecurity. Independent samples t-tests confirm that these cross-national differences are statistically significant in 10 of the 11 items analyzed, with Chile consistently displaying higher levels of concern.

Differences are particularly pronounced in items related to economic insecurity, such as the inability to pay bills, afford basic necessities, secure housing, or cover medical treatments. These findings suggest that concerns about economic vulnerability are more intense in Chile. In contrast, although levels of concern in Mexico are comparatively lower on average, certain items associated with social vulnerability—such as experiencing unfair treatment or violence—also show relatively elevated mean values in both countries. The only item that does not exhibit a statistically significant difference between countries is the experience of violence.

Overall, the results indicate that the positional inconsistency trial is perceived with greater intensity in Chile, especially in its economic dimension, whereas in Mexico perceptions tend to be comparatively less intense.

#### 4.1.3 Access to resources

Table 6 presents the mean scores, standard deviations, and mean differences for access to resources through social networks. The results show that family constitutes the primary source of material and caregiving support in both countries, while friendships play a complementary role.

**Table 6:** Access to resources through networks: means, standard deviations, and *t*-tests by country.

Item	Mexico mean	Mexico SD	Chile mean	Chile SD	*t*	*p*
If I needed money, a family member could lend it to me	4.32	0.99	3.78	1.25	4.24	< 0.001***
If I needed a vehicle, I could turn to a family member	4.29	1.09	3.72	1.34	4.05	< 0.001***
If I needed a vehicle, I could turn to a friend	3.51	1.29	3.25	1.32	1.71	0.088
If I or a household member suffered a serious illness, a family member would support us with care	4.40	0.92	3.88	1.09	4.41	< 0.001***
If I or a household member suffered a serious illness, a friend would support us with care	3.42	1.23	3.33	1.23	0.60	0.551
If needed, a family member or friend would help with childcare and/or elder care	3.91	1.15	3.76	1.18	1.13	0.258
If I did not have a place to sleep, a family member could host me temporarily	4.62	0.78	4.28	0.96	3.37	0.001**
If I did not have a place to sleep, a friend could host me temporarily	4.08	1.10	3.85	1.17	1.71	0.089

Scale ranges from 1 (Strongly disagree) to 5 (Strongly agree).

**p < 0.01; ***p < 0.001.

Source: authors’ elaboration, 2025.

Comparatively, Mexico displays systematically higher levels of perceived access to relational resources, particularly in indicators associated with family support, such as financial assistance, access to vehicles, caregiving during illness, and temporary housing. Independent samples t-tests confirm statistically significant differences in these family-related items (*p* < 0.01), with consistently higher mean values in Mexico.

In contrast, no statistically significant differences are observed in items referring to support from friends, where mean levels are relatively similar across countries. Overall, these findings indicate that access to relational resources is higher in Mexico and that, in both contexts, support structures are primarily organized around family ties.

#### 4.1.4 Social capital

As indicated in Section 3.1, social capital was measured using a position generator, which identifies access to specific occupations within respondents’ social networks. Table 7 presents the percentage of respondents reporting at least one contact in each occupational position, ordered by their score on the International Socio-Economic Index of Occupational Status (ISEI). In both countries, access spans a broad range of occupational statuses, although differences in frequency are observed across positions.

**Table 7:** Accessed occupational positions (ISEI score), percentage of respondents with at least one contact in their network.

Occupation	ISEI score	Mexico (%)	Chile (%)
Manager or director of a large company	88.0	82.4	57.1
Street vendor	85.0	63.9	43.8
Secretary	77.0	76.0	74.1
Auto mechanic	68.1	67.2	50.9
Store or shop clerk	55.1	63.8	68.8
Lawyer	54.0	89.9	76.8
Office cleaner	52.9	59.2	68.8
Physician	43.0	89.5	72.3
Preschool educator	40.5	77.4	66.1
Taxi driver	34.0	56.8	47.3
Waiter or assistant	34.0	45.8	42.9
Accountant	30.0	88.8	71.4
University professor	28.5	78.4	77.7
Nurse	16.0	65.6	74.1

Percentages represent respondents reporting at least one contact in their personal network occupying the specified position. Missing values were recoded as “no access” and are not reported separately.

ISEI, international socio-economic index of occupational status.

Source: authors’ elaboration, 2025.

Building on this information, two continuous indicators were constructed and are presented in Table 8: network diversity, defined as the number of occupational positions to which respondents have access, and network status, calculated as the average ISEI score of these positions.

**Table 8:** Social capital indicators: means, standard deviations, and *t*-tests by country.

Indicator	Mexico mean	Mexico SD	Chile mean	Chile SD	*t*	*p*
Network diversity	10.01	2.67	8.92	3.68	2.74	0.007**
Network status (ISEI)	38.34	9.11	33.76	13.67	3.18	0.002**

Higher values indicate greater network diversity and higher average occupational status of contacts. **p < 0.01.

Source: authors’ elaboration, 2025.

The results reveal statistically significant cross-national differences. In Mexico, network diversity is higher (Mean = 10.01; SD = 2.67) than in Chile (Mean = 8.92; SD = 3.68). Similarly, average network status is higher in Mexico (Mean = 38.34; SD = 9.11) compared to Chile (Mean = 33.76; SD = 13.67). Independent samples t-tests confirm that both differences are statistically significant (*p* < 0.01).

Taken together, Tables 7, 8 indicate that respondents in Mexico not only report access to a wider range of occupational positions, but also to contacts with higher average socio-economic status. In Chile, access appears comparatively more restricted and more heterogeneous.

#### 4.1.5 Social origin

Social origin was operationalized using the educational attainment of the mother and father, following the ISCED classification. In both countries, households with secondary and tertiary education predominate, although their distribution differs. In Mexico, the highest concentration is observed in complete secondary education, whereas in Chile there is a greater presence of incomplete secondary education alongside a similar proportion of tertiary education.

Intergenerational educational transitions show higher levels of upward mobility in Chile, reaching 78.6% relative to the mother and 73.5% relative to the father, compared to 72.8% and 60% in Mexico, respectively. Downward mobility is also less frequent in Chile, while Mexico exhibits higher proportions of educational decline. Overall, these findings reveal important differences in the structure of social origin and educational mobility patterns between the two countries (see Table 9).

**Table 9:** Social origin and intergenerational educational mobility (Mexico and Chile).

Indicator	Mexico		Chile	
	Mother	Father	Mother	Father
ISCED 1—incomplete primary	6.1%	2.5%	—	—
ISCED 2—complete primary	9.7%	7.6%	—	—
ISCED 3—incomplete secondary	2.0%	2.5%	21.4%	18.6%
ISCED 4—complete secondary	44.4%	40.4%	40.8%	39.2%
ISCED 5–8—tertiary education	37.8%	47.0%	37.9%	42.1%
Upward educational mobility	72.8%	60.0%	78.6%	73.5%
Educational immobility	20.0%	26.0%	18.0%	22.5%
Downward educational mobility	6.7%	14.0%	3.9%	—

Source: authors’ elaboration, 2025.

### 4.2 Latent variables (EFA and CFA)

#### 4.2.1 Exploratory factor analysis

Exploratory factor analysis was conducted using principal axis factoring with oblique Promax rotation, evaluating solutions ranging from one to five factors. Factor retention was based on eigenvalues, theoretical interpretability, and model fit indices. In both countries, the five-factor solution showed the best performance, with an RMSEA of approximately 0.066. In Chile, the five factors explained 51% of the total variance (17, 13, 8, 7, and 7%), while in Mexico they explained 43%.

The resulting structure was consistent with the theoretical dimensions of the instrument: (1) labor market strain, (2) positional inconsistency strain, (3) access to resources, (4) social capital, and (5) social origin. Moderate correlations among factors support the multidimensional nature of the construct.

Internal consistency was adequate. Standardized alpha reached 0.84 in Chile and 0.87 in Mexico. Omega coefficients indicated high composite reliability (G6 ≈ 0.93; ω_t_ = 0.88 in Chile and 0.91 in Mexico). Hierarchical omega values were lower (ω_h_ = 0.27 and 0.34), suggesting that common variance is not dominated by a single general factor, supporting the appropriateness of a multidimensional structure.

#### 4.2.2 Confirmatory factor analysis

A confirmatory factor analysis was conducted to evaluate the adequacy of the measurement model and the relationship between observed variables and their corresponding latent^3^ constructs. Given the ordinal nature of the indicators, the model was estimated using the Diagonally Weighted Least Squares (DWLS) estimator, which is appropriate for ordinal categorical variables.

For Mexico, the model was estimated using the complete dataset. As shown in Table 10, the model demonstrated very good fit to the data, with CFI = 0.971, TLI = 0.968, and RMSEA = 0.048, all within recommended thresholds (Browne and Cudeck, 1993). The SRMR was 0.082, indicating acceptable fit.

**Table 10:** Model fit indices for the confirmatory factor analysis.

Fit index	Mexico	Chile	Evaluation
CFI (comparative fit index)	0.971	0.951	Excellent/acceptable
TLI (tucker–lewis index)	0.968	0.946	Excellent/acceptable
RMSEA (root mean square error of approximation)	0.048	0.069	Very good/acceptable
SRMR (standardized root mean square residual)	0.082	0.101	Acceptable/acceptable

Fit indices for Chile were estimated using multiply imputed data and combined following Rubin’s rules.

Source: authors’ elaboration, 2025.

For Chile, due to the presence of missing data, the model was estimated using multiple imputation. Five imputed datasets^4^ were generated, and parameter estimates were combined following Rubin’s rules. The results indicate adequate overall model fit, with CFI = 0.951, TLI = 0.946, RMSEA = 0.069, and SRMR = 0.101. Although the SRMR slightly exceeds conventional thresholds, the overall pattern of fit indices supports acceptable model fit.

Standardized factor loadings were statistically significant and ranged from moderate to high in both countries, ranging from 0.585 to 0.981 in Mexico and from 0.464 to 0.993 in Chile, supporting the convergent validity of the constructs.

Given that the aim of the analysis is to examine the internal structure of the model within each national context, structural models were estimated separately for Mexico and Chile.

### 4.3 Structural model

#### 4.3.1 Analytical model

As outlined in the introduction, the analytical model specifies the hypothesized relationships between ascribed and achieved factors and the two structural tests. To this end, a model was developed to assess how social origin, education, social capital, access to resources, and occupational status influence positional inconsistency and the work trial Table 11.

**Table 11:** Standardized factor loadings of observed variables by country.

Latent variable	Observed variable	Mexico	Chile
Work trial	At work, I can freely express my opinions without fear of being dismissed	0.671	0.672
	There is equal and respectful treatment at work	0.748	0.604
	If I have a problem, I receive help from my supervisors	0.639	0.635
	I frequently talk with my coworkers about how to carry out the work	0.656	0.815
	If needed, I will receive help from my coworkers	0.836	0.899
	There is an atmosphere of trust among coworkers	0.812	0.839
	I feel that I am part of a work group or team	0.860	0.872
Positional inconsistency trial	You or a member of your family losing their job	0.708	0.799
	Not having a place to live in the near future	0.805	0.892
	Not being able to pay bills in the coming months	0.890	0.978
	Not having enough money to purchase necessary food	0.937	0.920
	Not having money to cover medical treatments	0.882	0.874
	Having to change your lifestyle due to financial constraints	0.824	0.675
	Becoming over-indebted	0.795	0.697
	Not being able to find jobs with better working conditions	0.652	0.665
	Experiencing unfair treatment	0.677	0.725
	Losing others’ esteem, recognition, and support	0.637	0.690
	Experiencing violence of any kind	0.667	0.721
Access to resources	If needed, a family member could lend me money	0.585	0.464
	If needed, I could rely on a family member for a vehicle	0.757	0.510
	If needed, I could rely on a friend for a vehicle	0.623	0.466
	If I or a household member becomes seriously ill, a family member would provide care	0.667	0.805
	If I or a household member becomes seriously ill, a friend would provide care	0.655	0.531
	If needed, a family member or friend would help with childcare or eldercare	0.614	0.667
	If needed, a family member could provide temporary housing	0.914	0.684
	If needed, a friend could provide temporary housing	0.641	0.634
Social capital	Network diversity	0.981	0.993
	Average occupational prestige of network contacts	0.981	0.993
Social origin	Mother’s educational attainment	0.840	0.731
	Father’s educational attainment	0.840	0.731

Source: authors’ elaboration, 2025.

**Figure 1:**
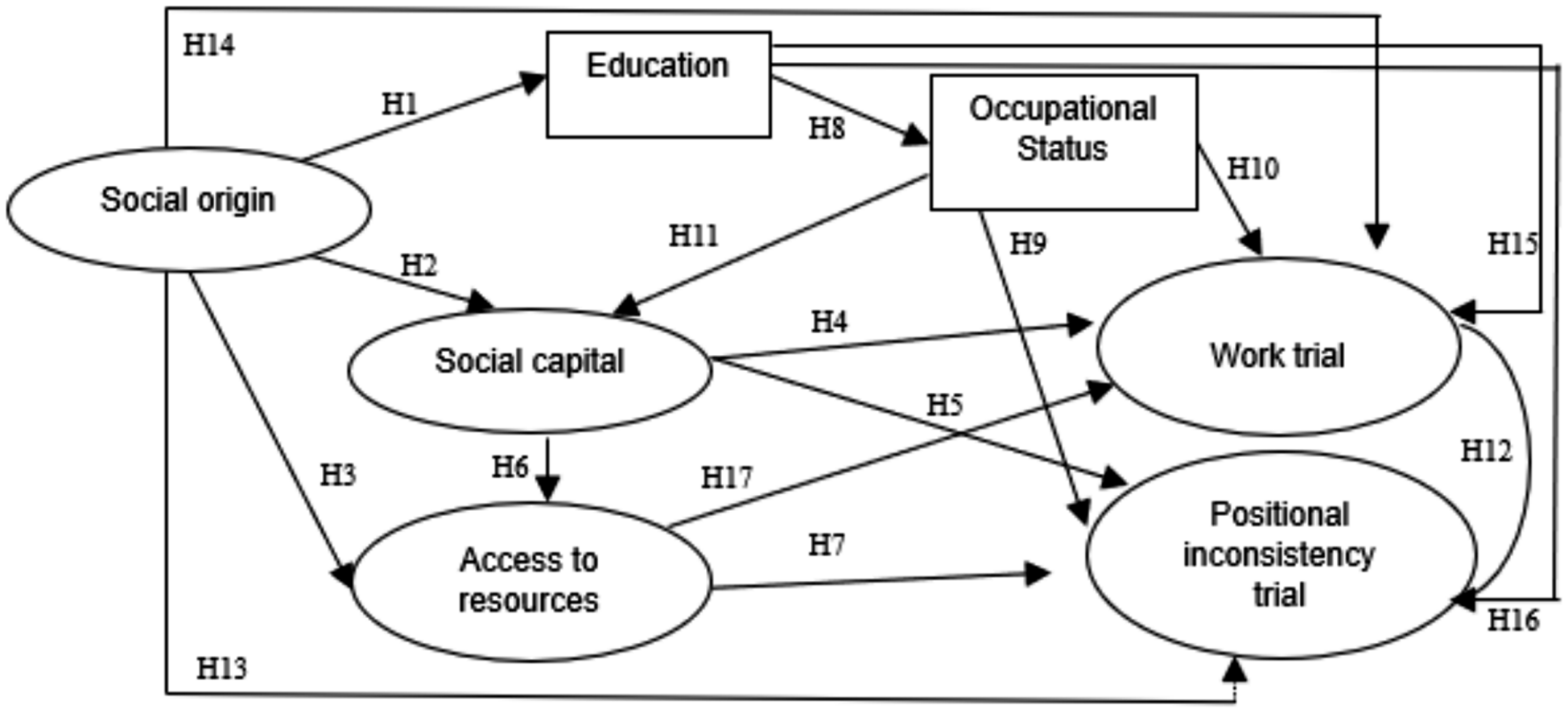
Analytical model. Source: authors’ elaboration, 2025.

For both countries—Chile and Mexico—the same structural model was specified, designed to empirically examine these relationships in highly unequal contexts. As shown in Figure 1, the model proposes that social origin, an exogenous variable, directly affects educational attainment (H₁), social capital (H₂), access to resources (H₃), positional inconsistency (H₁₃), and the work trial (H₁₄). In turn, educational attainment is expected to influence occupational status (H₈) as well as both structural tests (H₁₅ and H₁₆). Occupational status is assumed to be associated with social capital (H₁₁), positional inconsistency, and the work trial (H₉ and H₁₀). Likewise, social capital is expected to affect both tests (H₄ and H₅) and access to resources (H₆). Access to resources would be directly linked to both tests (H₇ and H₁₇). Finally, the two tests are also expected to be correlated (H₁₂).

#### 4.3.2 Empirical model for Mexico

**Figure 2:**
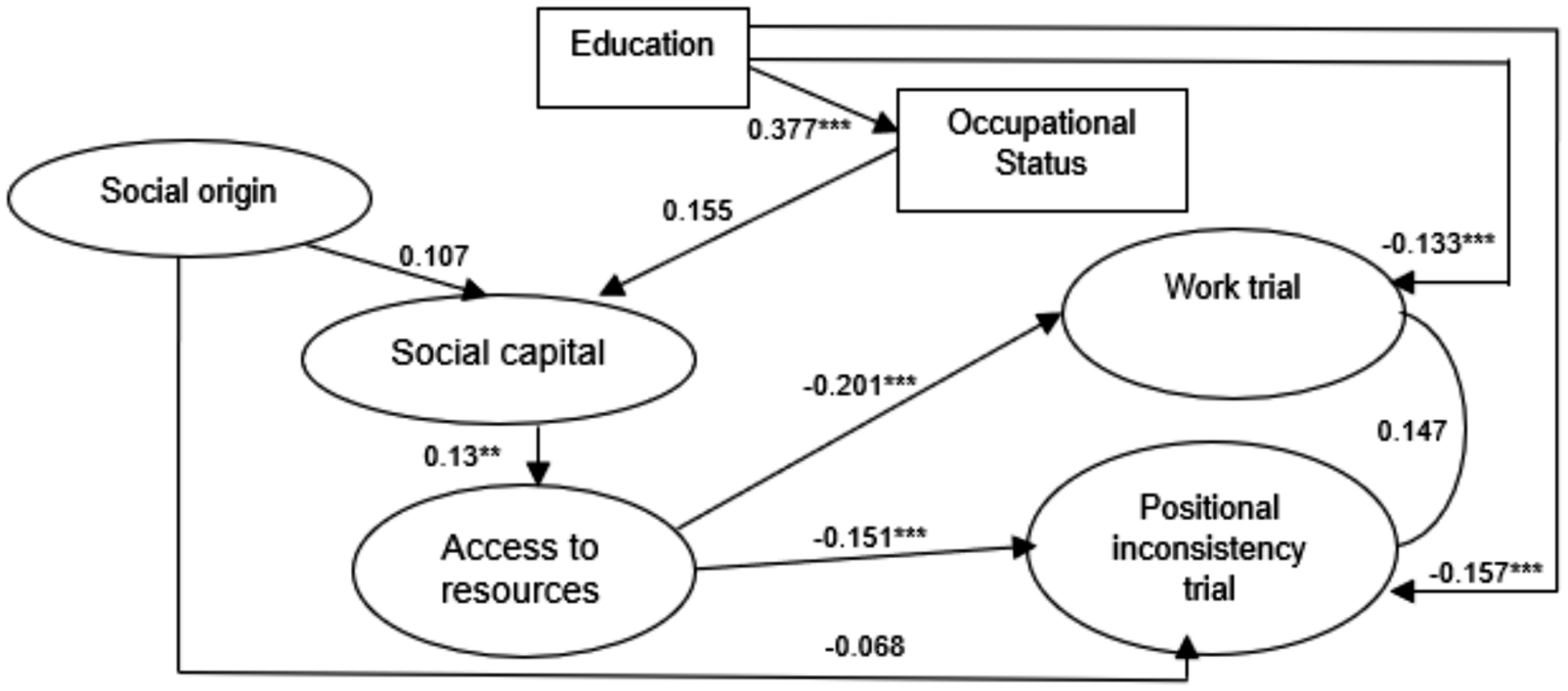
México model. Source: authors’ elaboration, 2025.

To examine the structural relationships in Mexico, a structural equation model was estimated following the analytical specification. The model showed an excellent overall fit to the data (CFI = 0.992; TLI = 0.992; RMSEA = 0.040; SRMR = 0.081) Figure 2.

Social origin showed a significant positive effect on social capital (H₂, *β* = 0.107, *p* < 0.05) and a significant negative effect on the work trial (H₁₄, *β* = −0.068, *p* < 0.05). No statistically significant effects of social origin were observed on educational attainment (H₁), access to resources (H₃), or the positional inconsistency trial (H₁₃), and these hypotheses were rejected.

Social capital was positively associated with access to resources (H₆, *β* = 0.139, *p* < 0.05), but showed no significant direct effect on the positional inconsistency trial (H₄) or the work trial (H₅).

Access to resources exerted a negative effect on both structural trials: positional inconsistency (H₇, *β* = −0.151, *p* < 0.05) and work trial (H₁₇, *β* = −0.201, *p* < 0.05).

Educational attainment positively affected occupational status (H₈, *β* = 0.377, *p* < 0.05) and also showed negative effects on positional inconsistency (H₁₅, *β* = −0.133, *p* < 0.05) and the work trial (H₁₆, *β* = −0.157, *p* < 0.05).

Occupational status exerted a positive effect on social capital (H₁₁, *β* = 0.155, *p* < 0.05) but showed no significant effects on positional inconsistency (H₁₀) or the work trial (H₉).

Finally, the positional inconsistency trial and the work trial were positively correlated (H₁₂, *β* = 0.14, *p* < 0.05).

#### 4.3.3 Empirical model for Chile

To examine the empirical adequacy of the model in the Chilean context, a structural equation model equivalent to that estimated for Mexico was specified. The model showed a satisfactory overall fit to the data (CFI = 0.987; TLI = 0.986; RMSEA = 0.077; SRMR = 0.098^5^).

**Figure 3:**
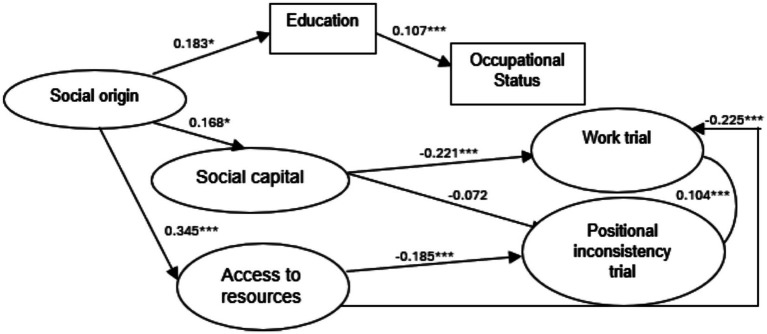
Chile model.

In Chile, social origin showed significant positive effects on educational attainment (H₁, *β* = 0.183, *p* < 0.05), social capital (H₂, *β* = 0.168, *p* < 0.05), and access to resources (H₃, *β* = 0.345, *p* < 0.05). However, no statistically significant direct effects of social origin were observed on the positional inconsistency trial (H₁₃) or the work trial (H₁₄), and these hypotheses were rejected Figure 3.

Social capital showed significant negative effects on the positional inconsistency trial (H₄, *β* = −0.221, *p* < 0.05) and on the work trial (H₅, *β* = −0.072, *p* < 0.05). By contrast, no statistically significant effect of social capital on access to resources was observed (H₆).

Access to resources showed significant negative effects on both structural trials. Specifically, access to resources negatively affected the positional inconsistency trial (H₇, *β* = −0.255, *p* < 0.05) and the work trial (H₁₇, *β* = −0.185, *p* < 0.05).

Educational attainment showed a positive effect on occupational status (H₈, *β* = 0.107, *p* < 0.05). However, educational attainment did not show statistically significant direct effects on the positional inconsistency trial (H₁₅) or the work trial (H₁₆).

Occupational status did not show statistically significant direct effects on the positional inconsistency trial (H₁₀) or the work trial (H₉).

Finally, a positive correlation was observed between the positional inconsistency trial and the work trial (H₁₂, *β* = 0.104, *p* < 0.05).

#### 4.3.4 Comparative summary of hypothesis testing

To facilitate cross-national comparison and provide a synthetic overview of the structural results, Table 12 summarizes the empirical status of hypotheses H₁–H₁₇ in Mexico and Chile. The table indicates whether each path is statistically significant and in the expected direction.

**Table 12:** Summary of hypothesis testing (H1–H17) in Mexico and Chile.

Path	Description	Mexico	Chile
H1	Social origin → Education	✖	✓ (+)
H2	Social origin → Social capital	✓ (+)	✓ (+)
H3	Social origin → Access to resources	✖	✓ (+)
H4	Social capital → Positional inconsistency	✖	✓ (−)
H5	Social capital → Work trial	✖	✓ (−)
H6	Social capital → Access to resources	✓ (+)	✖
H7	Access to resources → Positional inconsistency	✓ (−)	✓ (−)
H8	Education → Occupational status	✓ (+)	✓ (+)
H9	Occupational status → Work trial	✖	✖
H10	Occupational status → Positional inconsistency	✖	✖
H11	Occupational status → Social capital	✓ (+)	✖
H12	Correlation between tests	✓ (+)	✓ (+)
H13	Social origin → Positional inconsistency	✖	✖
H14	Social origin → Work trial	✓ (−)	✖
H15	Education → Positional inconsistency	✓ (−)	✖
H16	Education → Work trial	✓ (−)	✖
H17	Access to resources → Work trial	✓ (−)	✓ (−)

✓ indicates statistically significant effect (*p* < 0.05) in the expected direction; ✖ indicates non-significant effect.

Source: authors’ elaboration, 2025.

Taken together, the structural results reveal differentiated patterns in the mechanisms linking social origin, resources, and the two structural trials across countries. In Mexico, the model operates primarily through educational attainment and access to resources, both of which show consistent direct effects on positional inconsistency and the work trial. In Chile, by contrast, social origin exhibits a broader direct influence on educational attainment, social capital, and access to resources, while social capital plays a more central role in shaping both structural trials. In neither country does occupational status exert a direct effect on the trials, and no robust indirect effects were identified. Overall, the findings suggest that while the same analytical model is applicable in both contexts, the relative weight of educational, relational, and resource-based mechanisms differs, reflecting distinct configurations of inequality in each national setting.

#### 4.3.5 Indirect effects

No statistically significant indirect effects were observed in either country. In Chile, only one indirect path approached marginal significance (social origin → social capital → work trial), but it did not reach conventional levels of statistical significance. Overall, the findings indicate that the structural relationships identified operate primarily through direct effects rather than mediated pathways.

## 5 Discussion

### 5.1 Similarities and differences in the structural models

Overall, the structural models estimated for Mexico and Chile show a general structure consistent with the theoretical assumptions linking ascribed and achieved factors to exposure to structural trials. In both countries, access to resources and support networks operate as buffers against the positional inconsistency trial and the work trial, while educational attainment is systematically associated with occupational status. Moreover, the two structural trials are positively correlated, indicating that individuals tend to experience these challenges simultaneously.

Cross-national comparisons reveal significant differences in the strength and direction of effects, particularly regarding the role of social origin and the social mechanisms through which advantages are channeled. In Chile, social origin functions as a broad structural determinant, influencing educational attainment, social capital, and access to resources simultaneously. In Mexico, by contrast, its effect is more limited, concentrating mainly on social capital and, to a lesser extent, the work trial.

The effects of social capital also show notable contrasts. In Mexico, social capital does not directly affect the structural trials but operates primarily through access to resources. In Chile, however, social capital directly reduces the intensity of the work trial and the positional inconsistency trial, suggesting that the diversity and status of networks play a more active role in shaping perceptions of social position stability and work-related vulnerabilities.

Considering these differences in direct effects, indirect pathways were estimated to evaluate whether social origin influences the structural trials via intermediate variables, particularly social capital, occupational status (ISEI), and access to resources.

In Mexico, indirect effects were weak or non-significant, indicating that variables such as ISEI, social capital, or access to resources do not substantially mediate the relationship between social origin and the structural trials. The only pathway approaching an interpretable effect, though marginal, is social origin → social capital, consistent with literature highlighting the role of networks as a relevant mechanism for labor market insertion in contexts of occupational segmentation (Granovetter, 1973; Lin, 2001).

In Chile, indirect effects of social origin on the work trial and positional inconsistency trial were also close to zero and not statistically significant. Nevertheless, a marginally significant pathway emerged (*p* = 0.098; *β* = −0.038): social origin → social capital → work trial. This result indicates a partially negative mediation of social capital, where a more advantaged social origin is associated with more diverse and higher-status networks, reducing the perceived intensity of this trial. This finding aligns with literature emphasizing the ambivalent role of social capital in unequal contexts, functioning simultaneously as a resource for insertion and a mechanism for reproducing social hierarchies (Bourdieu, 1986; Lin, 2001).

In summary, the results indicate that the mechanisms through which social origin shapes exposure to structural trials vary across national contexts: in Chile, effects are broader and more direct, while in Mexico, influence primarily operates through relational resources, with limited indirect effects.

## 6 Conclusion

The analysis confirms that structural trials constitute a relevant analytical framework for understanding how inequality is experienced in Latin American contexts characterized by high levels of fragmentation and stratification. The cases of Mexico and Chile show that the tensions associated with the positional inconsistency trial and the work trial are not isolated phenomena, but rather embedded in everyday experiences where labor vulnerabilities, educational trajectories, and differentiated relational supports converge. Consequently, contemporary precarity does not take a homogeneous form; it is expressed according to the resources and ties that each society distributes and legitimizes.

The empirical models reveal a structure compatible with theoretical assumptions, although with notable cross-national variations. In Chile, social capital and access to resources—both influenced by social origin—directly affect perceptions associated with the trials. In Mexico, by contrast, only access to resources shows a direct relationship with one of the trials, while education plays a more determinative role as a protective mechanism against precarity. These differences can be understood in light of the trajectories of both educational systems: in Chile, the massification of access coexists with profound segmentation and credential devaluation, generating a pattern of educational mobility marked by inequality. In Mexico, lower absolute mobility and greater structural rigidity limit the scope for social origin to exert additional effects, which is reflected in the more circumscribed associations observed in the SEM models.

It is important to note several limitations of this study that should be considered when interpreting the results. The non-probabilistic, convenience-based sample restricts external validity and prevents generalization to the broader population. These characteristics also hinder comparisons with other studies. Nevertheless, the work provides a valuable approach for studying social stratification by incorporating a relatively unexplored analytical perspective and operationalizing it through structural equation models. Within this framework, it is important to acknowledge that not all estimated effects were statistically significant. However, statistical significance alone does not determine the plausibility or scientific relevance of an effect; distinguishing between statistical inference and scientific inference remains crucial for adequately interpreting the findings (Wasserstein et al., 2019).

A relevant avenue for future research is the formal evaluation of multi group invariance across countries. Although this study includes descriptive mean comparisons between Chile and Mexico, the structural equation models were estimated and analyzed separately for each country, as the objective was not to directly compare coefficient magnitudes but to examine the internal configuration of relationships within each national context. In this regard, the absence of metric and scalar invariance tests limits the ability to make strictly structural cross-national comparisons, but does not compromise the validity of within-country analyses. Future studies with probabilistic samples and greater statistical power could incorporate multi group invariance analyses to more accurately assess construct equivalence and strengthen comparative inferences.

Despite these limitations, the findings collectively indicate that understanding vulnerabilities across Latin American trajectories requires an approach that considers not only the positions individuals occupy but also how each society mobilizes and distributes relational resources. The results reveal that, while Chile and Mexico share patterns of structural inequality, the mechanisms through which this inequality is experienced, interpreted, and addressed differ. This comparison not only highlights national specificities but also contributes to a more precise theoretical understanding of how structural trials operate in unequal contexts, encouraging the conceptualization of inequality as a situated, relational, and dynamic experience.

Finally, extending the study to more heterogeneous samples and comparing it with findings from other countries would be valuable to explore common and divergent patterns in diverse socio-cultural contexts. This would allow for a more nuanced understanding of structural trials and a deeper exploration of the specific forms that inequality takes.

## Data Availability

The raw data supporting the conclusions of this article will be made available by the authors, without undue reservation.

## Footnotes

1 To compare parents’ educational attainment in Mexico and Chile, the ISCED (International Standard Classification of Education) classification was used, which harmonizes educational systems into five equivalent levels.

2 Although no explicit measure of social class is included, the inclusion criteria—income level, occupational position, and metropolitan residence—allow the sample to approximate urban middle-class sectors.

3 It is important to note that, following the pilot phase, data collection in Mexico was conducted using a longer questionnaire than the one administered in Chile. The implementation of the Mexican instrument made it possible to examine the initial factorial structure, refine the items, and estimate a preliminary SEM model. Consequently, the model applied to the Chilean sample was not developed from scratch but was based on the structure previously identified during the Mexican phase.

4 The imputation was performed using the *mice* function in R, through which five imputed datasets were generated.

5 In the case of Chile, the model was estimated using a database with imputed values to handle missing data; the fit indices correspond to this specification.

## Author notes

This article is part of the doctoral thesis “The Social Bond in Societies with Close Relational Supports: The Cases of Chile and Mexico,” carried out within the PhD Program in American Studies at the University of Santiago de Chile. The research addresses the configurations of the social bond and the structural challenges in Latin American contexts, with an emphasis on the cases of Chile and Mexico. The present text represents a partial synthesis of the theoretical and empirical findings that will be further developed in the final version of the thesis.
